# RNA-protein distance patterns in ribosomes reveal the mechanism of translational attenuation

**DOI:** 10.1007/s11427-014-4753-8

**Published:** 2014-10-18

**Authors:** YU DongMei, ZHANG Chao, QIN PeiWu, CORNISH V. Peter, XU Dong

**Affiliations:** 1 Department of Biological Engineering, University of Missouri, Columbia, MO 65211, USA; 2 C.S. Bond Life Science Center, University of Missouri, Columbia, MO 65211, USA; 3 Department of Computer Science, University of Missouri, Columbia, MO 65211, USA; 4 Department of Biochemistry, University of Missouri, Columbia, MO 65211, USA

**Keywords:** ribosome, protein translation, antibiotics, translocation, RNA-protein interaction

## Abstract

Elucidating protein translational regulation is crucial for understanding cellular function and drug development. A key molecule in protein translation is ribosome, which is a super-molecular complex extensively studied for more than a half century. The structure and dynamics of ribosome complexes were resolved recently thanks to the development of X-ray crystallography, Cryo-EM, and single molecule biophysics. Current studies of the ribosome have shown multiple functional states, each with a unique conformation. In this study, we analyzed the RNA-protein distances of ribosome (2.5 MDa) complexes and compared these changes among different ribosome complexes. We found that the RNA-protein distance is significantly correlated with the ribosomal functional state. Thus, the analysis of RNA-protein binding distances at important functional sites can distinguish ribosomal functional states and help understand ribosome functions. In particular, the mechanism of translational attenuation by nascent peptides and antibiotics was revealed by the conformational changes of local functional sites.

Super-molecular complexes are composed of multiple components that perform biological functions in a coordinated fashion. The ribosome is one such super-molecular complex with more than 50 proteins and three strands of RNA [1]. The bacterial 70S ribosome (2.5 MDa) consists of two subunits: 30S small subunit and 50S large subunit [2]. The ribosome has three tRNA binding sites: A, P, and E sites, and tRNA is accommodated onto the A site, translocated to the P site, and released at the E site. Eukaryotic 80S ribosomes (4 MDa) are composed of a 40S small subunit and a 60S large subunit. The first high-resolution X-ray crystal structure of a eukaryotic ribosome was resolved in 2010 [3], which is about 10 years later after the first publication of the bacterial ribosome structure [2]. The function of the ribosome is to translate the genetic code in mRNA into proteins through several distinct stages: initiation, elongation, and termination. In each cycle of elongation, one amino acid is added to the C-terminus of the newly synthesized peptide [4]. The ribosome associates and dissociates with different molecules in the process of translation, and the global and local structures of ribosome complexes change to perform different functions during different stages. Ribosome inter-subunit rotation (counterclockwise rotation between the two subunits) is a global conformational change that occurs during elongation, forming a rotated state [5].

Ribosomes exist in many different functional states, some of which appear in the PDB [2]. The structures of these ribosome complexes provide hints on the mechanism of translation initiation, translocation and peptidyl transfer [6]. An overlay of all the deposited ribosomes or ribosomal rRNAs indicates diverse conformational states. It is important to understand the local/minor changes that contribute to these large-scale changes. How then can a computational approach contribute to our understanding and how can this facilitate our ability to use this information for predicting the functional site of ribosome? In this study, we conducted a computational analysis on all the known ribosome complex structures, which, to our knowledge, represents the first comparative study among many ribosome complex structures. For this purpose, RNA-protein distances of ribosome complexes were collected based on the coordinates of ribosome structures in the PDB. The changes of RNA-protein binding distance among ribosome complexes can show the local conformational changes more easily. Analysis of RNA-protein or protein-protein distance in these ribosome complexes could provide insight into the function of the ribosome and the relationship between conformational changes and ribosome functions.

There are many functional sites in the ribosome such as the mRNA entrance tunnel, tRNA binding sites, initiation factor binding sites, elongation factor binding sites, and release factor binding sites [7,8]. The conformational changes of these local microenvironments are critical for the functions of ribosome complexes. The RNA-protein distances change when the ribosome adopts different functional states. The amplitude of local changes also can be easily observed by the degree of the data spread when we compare two ribosome complexes.

Protein translation is a highly regulated process. The rate of protein translation is tuned in part by the gene sequence [9]. Protein translation can also be interrupted by the binding of various factors. Antibiotics can attenuate the translation rate, and the degree of attenuation depends on the chemical composition and binding position of antibiotics. In addition, specific translated nascent peptides, truncated or defective mRNA, or mRNA structures can attenuate or inhibit protein translation and stall the ribosome. The cell has corresponding restoring systems to resurrect protein translation and rescue the stalled ribosomes. For example, the tmRNA-SmpB system can rescue the stalled ribosome by defective or truncated mRNA [10]. Ribosomes stalled by nascent peptides can be restored by protein export machinery [1]. The study of RNA-protein distance in these stalled or attenuated ribosomes in contrast to unrotated ribosomes can map the conformational changes of stalled ribosome and reveal the mechanism of translation attenuation.

## 1 Materials and methods

One hundred and six ribosome 3D structures (202 entries) from two species were collected from the PDB and were divided into four groups based on the ribosome source and subunit type. Table 1 shows part of above PDB entries used in this study *Escherichia coli* (*E. coli*) and *Thermus thermophilus* (*T. thermophilus*) as the examples. All PDB files used in this study and computational results can be found at http://digbio.missouri.edu/ribosomes/.

Due to missing coordinates and some inconsistent information in different PDB files, our measurements cannot be applied to raw PDB files directly; thus, we used the following pre-processing steps to retrieve information from each PDB entry:

- Parse the header of each PDB file to map chain ID and protein ID of each ribosomal protein, since the same protein might have different chain IDs in different PDB entries.
- Generate a protein list containing 30 proteins for the 50S and 19 proteins for the 30S. For each protein in the list, extract its coordinates and amino acid sequence.
- For the 50S large subunit, use coordinates of 5S rRNA and 23S rRNA, and combine them together; for the 30S small subunit, use only coordinates of 16S rRNA for rRNA structural information.
- For each ribosomal protein, apply multiple structural alignment (MSA) to structures from the same species by using the CE_MC server [11], which will allow an alignment profile to be generated.
- In each alignment profile, replace those amino acids with missing coordinates with gaps.

The pre-processed results were put into corresponding groups, according to the subunit and species information. Within each group, for one ribosomal protein, we calculated distances between all atoms of one particular amino acid and all atoms of all nucleotides of rRNA to find the minimum value as the distance between this particular amino acid and rRNA. In order to compare the RNA-protein distances between different ribosomes, these minimum distance values have been saved into one matrix with the same position/index as the corresponding alignment profile. The distance value of a gap in this alignment file was set as 0. All subsequent analyses and comparisons are based on the above calculating results.

## 2 Results and discussion

### 2.1 Comparison of ribosomes between *E. coli* and *T. thermophilus*

**Figure 1:**
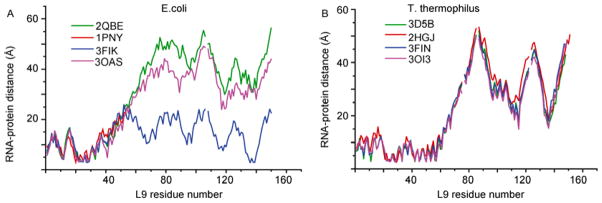
RNA-protein distance plot for ribosome protein L9 on the large subunit in *E. coli* (A) and *T. thermophilus* (B) ribosomes. The PDB codes where the ribosome structures were derived are labeled in the upper left corner.

The studies of ribosomes of these two bacteria during the past several decades have provided valuable insight to better understand ribosome functions at the molecular level. Ribosomes trapped at different stages of translocation or bound with different molecules have been resolved for both types of ribosomes [6,8,12,13]. Comparison of the RNA-protein distance patterns for *E. coli* and *T. thermophilus* ribosomes easily reveals the differences in mobile parts of the two ribosomes. The RNA-protein distance variation of L9 in different *E. coli* and *T. thermophilus* ribosome complexes were compared, where the *E. coli* and *T. thermophilus* ribosome complexes have similar binding molecules. The distance of L9 to 23s rRNA changes dramatically among *E. coli* ribosomes and very slightly among *T. thermophilus* ribosome complexes (Figure 1). L9 of the large subunit binds the domain V (nucleotide 1999–2776) of 23S rRNA [14].

The sequence identity between *T. thermophilus* and *E. coli* ribosome proteins are relatively low on average (Table 1). However, *T. thermophilus* ribosomes show similar RNA-protein distance pattern or a similar fold among the PDB entries. In general, RNA-protein distances for *T. thermophilus* ribosomes are slightly smaller than those of *E. coli*, which may result from the thermal stability of ribosomes. *E. coli* grows optimally at 37°C, while *T. thermophilus* grows optimally at 78°C and cannot survive if the temperature is below 50°C [15]. Another possible reason might be the “freezing effect” of crystallography since *T. thermophilus* function at high temperature and crystallize at room temperature. The comparison shows the structural similarity/difference between *T. thermophilus* and *E. coli* ribosomes. The local dissimilarity accounts for the selective action of antibiotics, which excludes the possibility of comparing two ribosomes directly. Thus, the antibiotics bound ribosome was analyzed only for *T. thermophilus* since there are more structures resolved than *E. coli*. The stalled ribosomes were analyzed only for *E. coli* since no stalled ribosome complexes are available for *T. thermophilus*.

### 2.2 Mechanism of macrolide antibiotics inhibition

**Figure 2:**
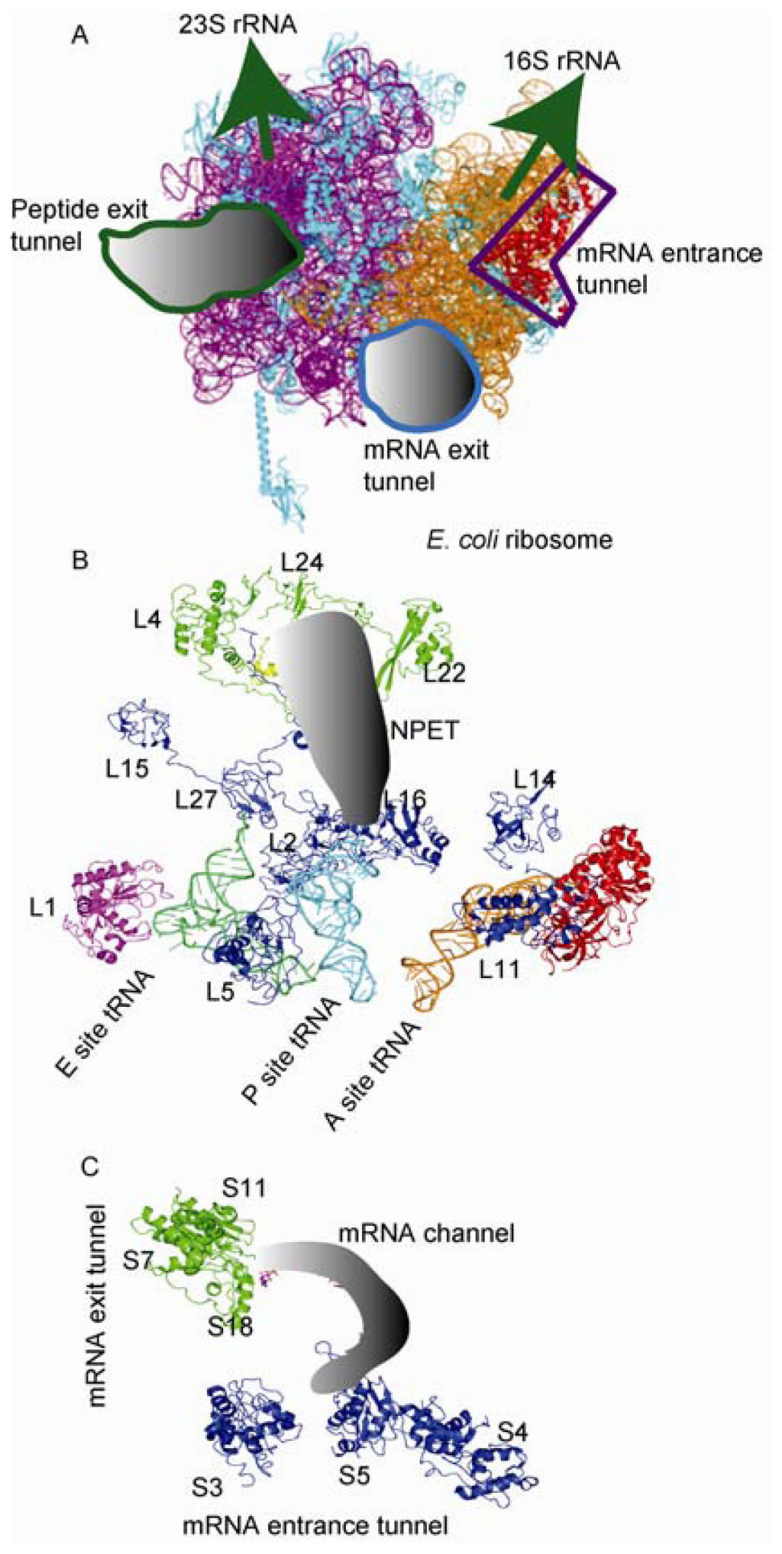
Ribosome functional sites. The ribosome proteins at mRNA entrance tunnel, mRNA exit tunnel, NPET, tRNA binding site are labeled. A, Ribosome model (based on 3R8N). B, NPET (labeled by red) and tRNA location on the large subunit (based on 3FIK). C, mRNA channel (labeled by red) (based on 3FIH).

Macrolide antibiotics are one of four important clinical families, which bind to the 23S rRNA at the upper third of the nascent peptide exit tunnel (NPET) and attenuate the growth of nascent peptides [16]. Proteins L4, L22, and L24 function near the NPET while L23 and L29 have been shown to be in close proximity to NPET (Figure 2) [17]. L4 and L22 protrude into the tunnel and RNA moieties form the narrowest part of the channel [1]. The length of the tunnel is about 90 Å in bacteria and 100 Å in eukaryotic ribosomes. However, the diameter of NPET is the same independent of the source; the diameter of NPET is about 15 Å at the upper third part, 10 Å in middle part and 25 Å at the exit. The narrowest part is formed by the β loop of L4 and L22 [1].

**Figure 3:**
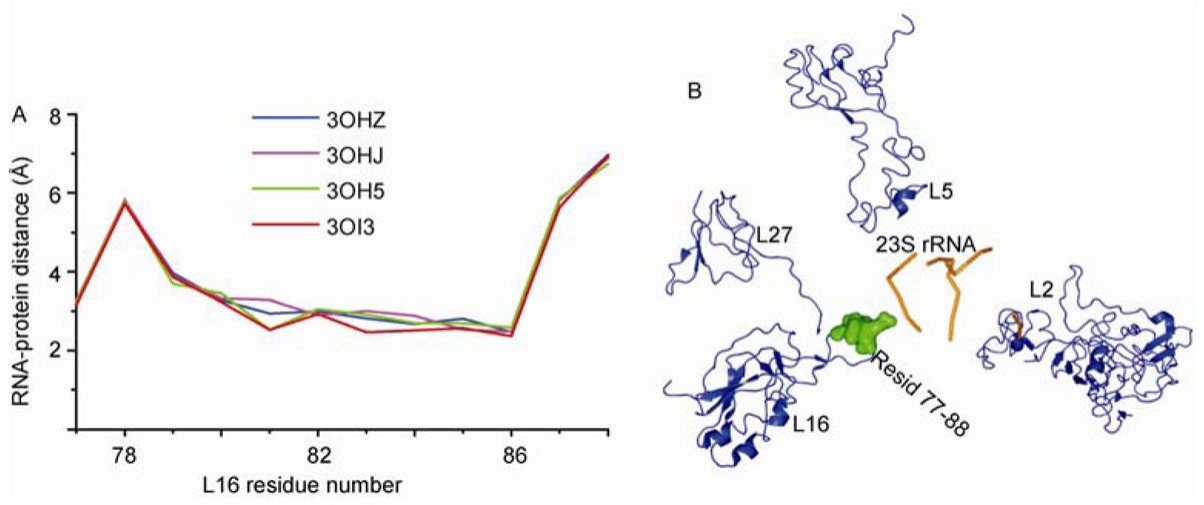
L16-RNA interaction. A, RNA-protein distance plot for ribosome protein L16 that is close to the macrolide-binding site. The PDB codes where the ribosome structures were derived are labeled. All the ribosomes shown are from *T. thermophilus.* B, Macrolide-binding site was colored in orange and the proteins near the binding site are shown.

Ribosome protein L16 is close to the macrolide-binding site of the 23S rRNA and the L16-rRNA interaction can modulate the local microenvironment for drug binding. L16 accommodates aminoacyl-tRNA at the A site [16]. The RNA-protein distance for ribosomes assembled with azithromycin (3OHZ), erythromycin (3OHJ), chloramphenicol (3OH5), and teliothromycin (3OI3) were compared (Figure 3). Chloramphenicol belongs to the phenicol family, whereas the other three antibiotics are from the macrolide family. Teliothromycin has a larger substitute group that is attached to the core ring structure. Erythromycin and azithromycin have the smaller substituent groups. For antibiotics (3OI3) with larger substituent groups, the RNA-protein distance at the binding site is smaller, which leaves more space for drug binding (Figure 3). Amino acid identity in the binding pocket determines the binding of antibiotics since mutations in the binding pocket make the bacteria resistant to antibiotics [16]. However, the local RNA-protein interaction can change the orientation, conformation, and exposure of functional groups in the binding site, which contributes to binding of different antibiotics and tunes the binding affinity. Positively charged residues K80 and K82 contribute to the interaction with negatively charged rRNA. The residues close to K80 and K82 are G79, G81, and G83. The absence of a side chain for glycine provides more space for free motion of positively charged residues. Antibiotics bind to the rRNA and block the NPET, which cause ribosome stalling and translation attenuation.

### 2.3 Translational attenuation by nascent peptides

The ribosome NPET provides a unique environment for nascent chain folding and a discriminating gate by blocking the translation of some specific proteins. However, some nascent peptides interact with 23S rRNA in the NPET and arrest translational elongation [18]. The ribosome stalled by a newly synthesized N-terminal peptide of TnaC shows dramatic conformational changes indicated by the large RNA-protein distance changes for most ribosomal proteins. In *E. coli*, the trigger factor is the first chaperone that interacts with the nascent polypeptides as soon as it emerges from the exit tunnel of the ribosome. Protein folding takes place only partially within this tunnel because the diameter is too narrow (10–20 Å) to accommodate tertiary folded proteins [17]. The tunnel is occluded in all of the stalled ribosome complexes [18].

**Figure 4:**
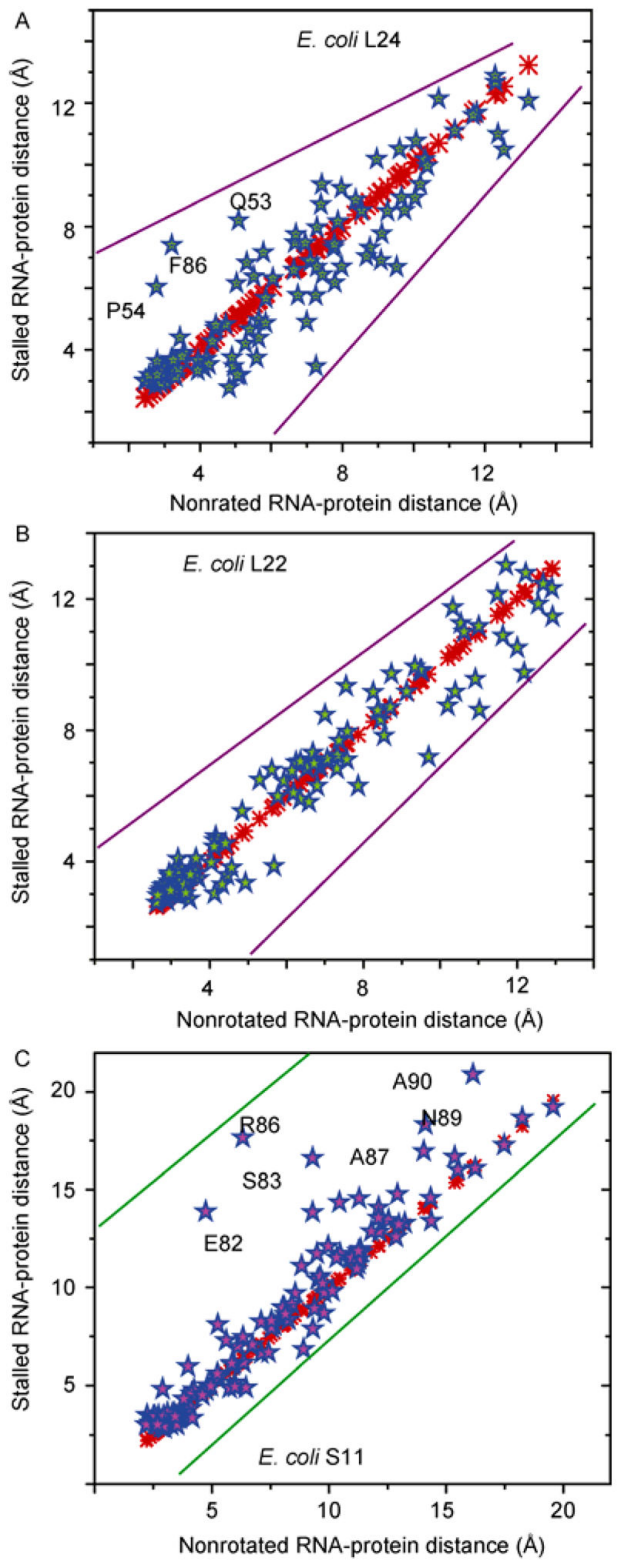
RNA-protein distance scatter plot of L24 and L22 at the peptide exit tunnel and S11 on mRNA exit tunnel for stalled (2WWQ) and unrotated ribosome (3R8T). Red crosshair represents the self-correlation of ribosome protein in the unrotated state. Blue, green and purple stars show the correlation of ribosome proteins L24, L22, and S11 in unrotated and stalled ribosome, respectively.

Figure 4 compares the RNA-protein distance changes between the stalled and unrotated ribosome in the unrotated state, where the inter-subunit did not rotate. The RNA-protein distances of the same proteins for the stalled and unrotated ribosomes indicate large conformational changes for proteins in the peptide and mRNA exit tunnel, which suggests that stalled ribosomes represent an unusual conformation that may lead to the dissociation of the large and small subunits if the translation cannot be resumed by recruiting protein factors like SRP and the trigger factor to the NPET. The large RNA-protein distances for both the large and small subunit proteins demonstrate the allosteric regulation of ribosome translation where the presence of a nascent translated peptide at the NEPT influences the proteins at the mRNA entrance tunnel, which is relatively far away (110 Å). Simultaneous interference of multiple functional sites can inhibit translation more efficiently than a single functional region. Allosteric regulation integrates the function of the large and small subunits. The overall changes of RNA-protein distance support the rearrangement of rRNA conformation during a ribosome stall.

**Figure 5:**
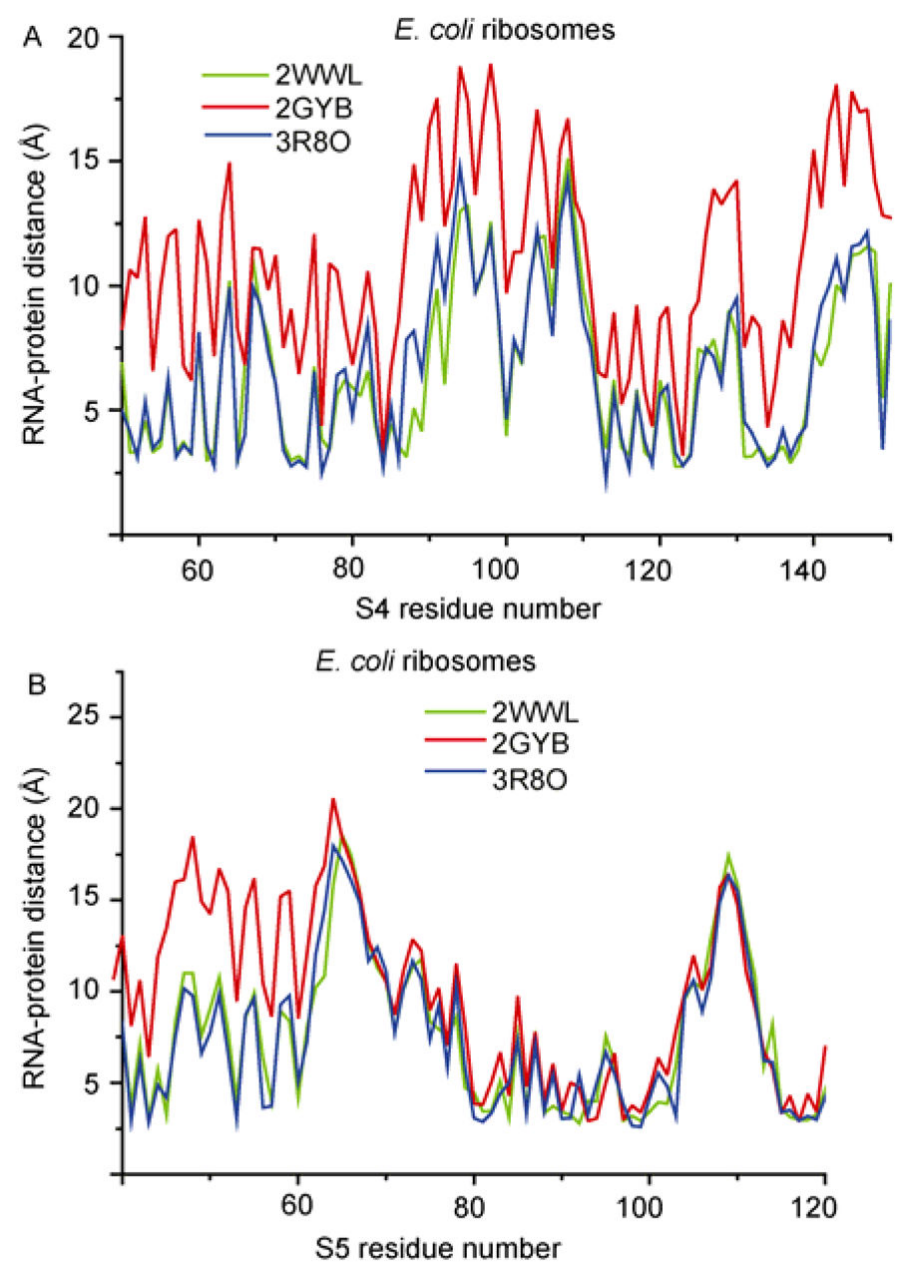
RNA-protein distance for proteins at mRNA entrance tunnel. A, RNA-protein for S4 is stalled by TnaC (2WWL), SecM (2GYB) and unrotated ribosome (3R8O). B, RNA-protein for S5 is among the stalled ribosome by TnaC (2WWL), SecM (2GYB) and unrotated ribosome (3R8O).

Ribosome stalled by different peptides or antibiotics show a different magnitude of conformational change. The amplitude of conformational changes reflects the strength of the stall. SecM induces larger-scale conformational changes in contrast to TnaC (Figure 5). The N-terminal stalling portion of SecM can be removed from the NPET of the stalled ribosome with SRP [18]. Current structural studies only resolve a limited number of stalled ribosome complexes. Thus, it is still difficult to predict the relationship between the nascent peptide sequence and the amplitude of conformational changes or the strength of the stall. Both the nascent peptides of TnaC and SecM block the NPET. However, the larger increase of RNA-protein distance for proteins at the mRNA entrance tunnel indicates that the mRNA entrance tunnel is also partially closed. Ribosomes stalled by nascent peptides are triggered by newly synthesized peptides in NPET, and the effect of the stall is propagated throughout the whole ribosome. Note that RNA-protein distance is different from the regular translation intermediates. Hence, the unusual conformation of the stalled ribosome suggests the nature of the correlation between different functional sites, where the modification of one functional site can interfere with the conformation of others. The local conformational changes at the NPET induce global 23S rRNA rearrangement and this rearrangement is propagated to the small subunit 16S rRNA through inter-subunit interactions. Protein conformations of the small subunit are adapted to the induced 16S rRNA conformational change.

**Figure 6:**
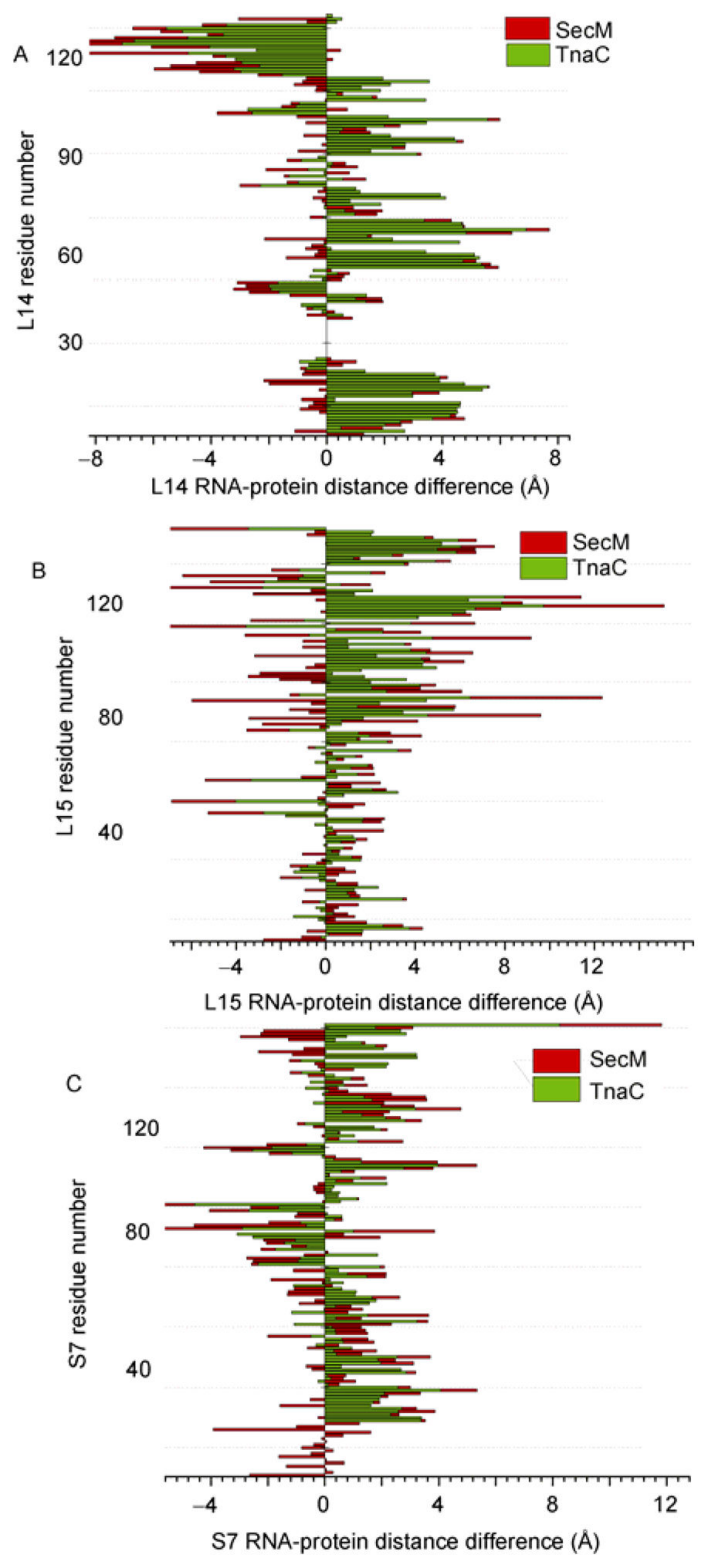
Comparison of RNA-protein distances. A, RNA-protein for L14 is stalled by TnaC (2WWQ) and SecM (2GYC). B, RNA-protein for L15 is stalled by TnaC (2WWQ) and SecM (2GYC). C, RNA-protein for S7 is among the stalled ribosome by TnaC (2WWL) and SecM (2GYB).

tRNAs associate with the proteins and rRNAs on both the large and small subunits, which also can function as a bridge for conformational signal transfer. Stalled ribosomes by a pseudoknot structure has shown the bending of tRNA [19]. The twisted tRNA will perturb the local conformation and RNA-protein distance for proteins on both the large and small subunits. Figure 6 plots the RNA-protein distance differences between the stalled and unrotated ribosomes against the residue number. For protein L15, the C-terminal domain of the protein moves close to the 23S rRNA shown by the negative RNA-protein distance difference (Figure 6A). For protein S7, residues 70–90 move close to the 16S rRNA while most of other parts of the protein move further away from rRNA (Figure 6B). Many proteins function at or near the tRNA binding site. Five proteins on small subunit, S7, S9, S12, S13, and S11, have active site contacts to the tRNA binding site. S9 and S13 contact the P site tRNA and are close to the decoding center. S7/S11 binds tRNA at the E site and S12 contacts tRNA at the A site. Ten proteins on the large subunit, L1, L2, L5, L11, L14, L16, L15, L26, L27, and L33 are located at or near the tRNA binding sites (Figure 2) [20]. L5 is located at the P site tRNA binding site and L16 is close to the A site. L33 interacts directly with the CCA end of the E site tRNA (Figure 2) [8]. tRNAs on the A and P sites communicate through a protein rich environment via the bridge formed by protein L31 [20]. The complex interaction of tRNAs with proteins on the large and small subunits lead to the role of tRNA in structural rearrangement of the whole ribosome. Comparison of RNA-protein distances for proteins near the tRNA binding site of stalled and unrotated ribosomes reveal the conformational changes of these proteins, which suggests that tRNA may be the allosteric bridge communicating the functional sites on both the small and large subunits.

### 2.4 Resumption of translation by interaction with SRP or other rescuing systems

**Figure 7:**
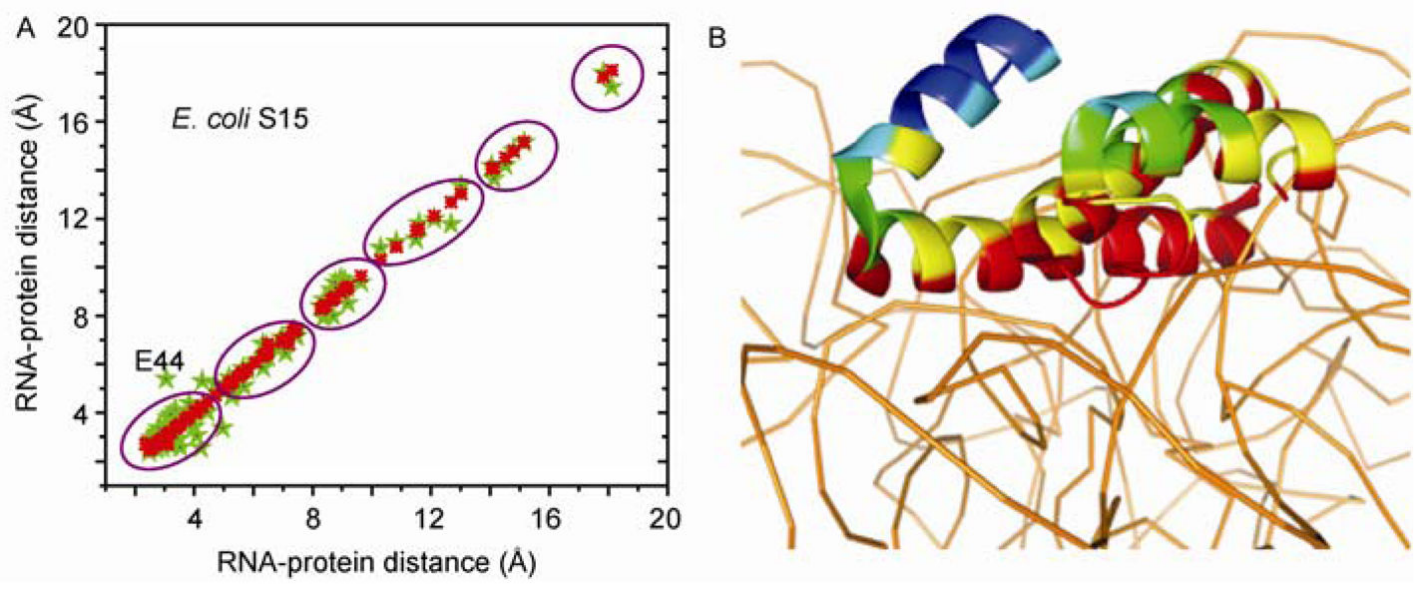
Distance correlation scatter plot of S15 and structural model of S15 on 16S rRNA. A, The block pattern of RNA-protein distance correlation. The two compared ribosomes were derived from PDB 3R8N and 3R8O. B, The residues of S15 (based on 3R8N) are labeled according to the RNA-protein distance. Red is less than 5 Å, yellow is between 5 and 7.5 Å, green is between 7.5 and 10 Å, and cyan is between 10 and 12 Å and blue is more than 12.5 Å. Green stars are residues of S15 from rotated ribosome and red dots are residues of 15 from unrotated ribosome.

Some ribosome proteins stay on the surface of the rRNA, which can be manifested in the block pattern of RNA-protein distance correlation scatter plots, where the RNA-protein distances from two ribosome complexes were plotted as the vertical and horizontal axis, as shown in Figure 7. The presence of a block pattern results from the hierarchical distribution of different layers of ribosomal proteins on the surface of rRNA. The localization of some ribosomal proteins on the surface makes them exposable to cytoplasmic regulators and mediates the communication between the ribosome and these regulating factors. For example, L23 binds rRNA and is close to the peptide exit tunnel. However, L23 is the central anchoring point for the SRP and trigger factor [18]. The proteins L24 and L29 also make contact with the SRP complex involved in the export of new proteins into and/or through cellular membranes [7]. Trigger factor binds the external globular domains of L29 and rRNA through a few contacts [21]. L10 (yeast) protein binds with NMD3 protein which is a nuclear export factor of the 60S subunit [22]. It is anticipated that more molecules can associate with ribosomal proteins transiently because weak protein interactions are difficult to identify using traditional methods [23].

The study of protein conformation and RNA-protein distances for proteins on the surface can help reveal potential targets for regulating factor binding since ribosome proteins bind regulatory proteins in a transient and reversible manner. RNA-protein distance changes for proteins on the surface can reveal the exposure of functional groups and binding properties of these proteins. A shorter RNA-protein distance indicates that the residues are in contact with rRNA and may be less likely to be used for binding with other molecules. A larger RNA-protein distance indicates the residues are more likely to be involved in interaction with other factors. Also, the number of protein residues and the ratio of residues in close contact (≤ 3.5 Å) with rRNA to the total residues represent the contact area between RNA and protein. The database for protein or ligand binding sites has been developed [24], which can be combined with RNA-protein distance analysis to map the potential binding sites on the ribosome surface.

**Figure 8:**
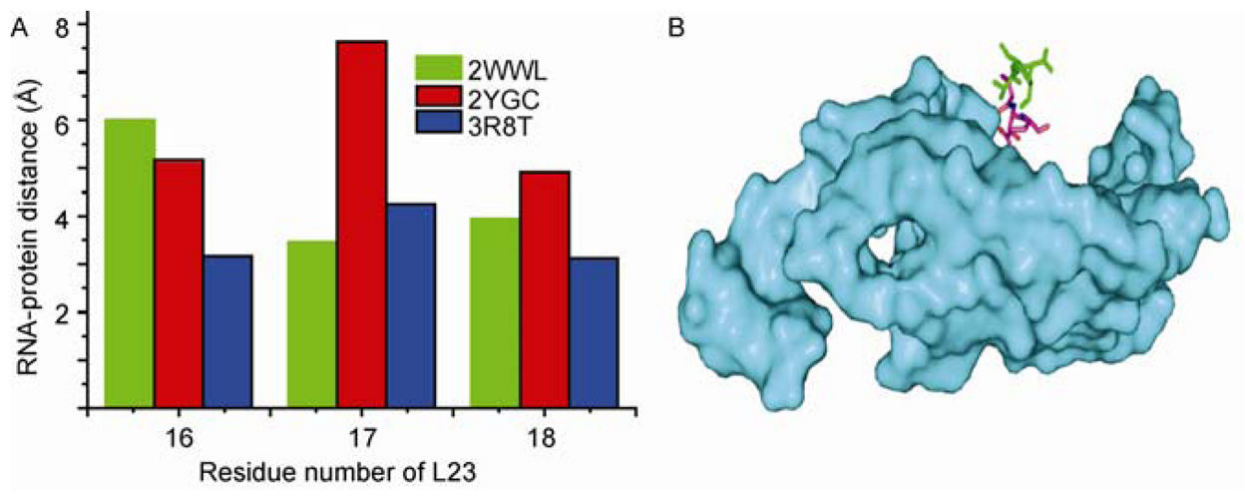
RNA-protein distance comparison and the model of distance change on the structure. A, RNA-protein distances for residues 16–18 of L23 are compared between stalled ribosome (2WWL for TnaC stall and 2YGC for SecM stall) and unrotated ribosome (3R8T). B, RNA-protein distance on the structure model. The green stick is the residues 16–18 of L23 in SecM stalled ribosome and the purple stick represents residues 16–18 of L23 in the unrotated ribosome.

Stalled ribosomes can be rescued by different mechanisms dependent on the cause of the stall. SecM is a bacterial secretory protein and the nascent translated peptide of SecM inhibits translation. The stalling peptide can be recognized by SRP and lead to transportation to the membrane Sec machinery for translocation across the cell membrane [18]. Trigger factor also can bind SRP complex and is excluded from the complex by SRP binding to the SRP receptor. Both SRP and trigger factor bind to L23. Which raises the question, how do SRP and trigger receive the signal that the ribosome was stalled? We hypothesize that the exposure of SRP or trigger factor binding sites indicates the stalled state of ribosome. Cross-linking studies have shown that residues 16–18 of L23 are critical for the binding of trigger factor. Thus, comparison of the local RNA-protein distance changes of L23 between stalled and unrotated ribosome can reveal the conformational change of the binding pocket (Figure 7A). For SecM stalled ribosome, the trigger factor to binding site has larger rRNA-protein distance, which exposes the residues in this region for trigger factor binding. The change of residues 16–18 relative to the 23S rRNA surface is shown (Figure 8B).

### 2.5 Translational attenuation by mRNA or truncated mRNA

Ribosome translation can be attenuated by rare mRNA codons for a particular protein and stalled ribosomes can be purified by immunoprecipitation. The mRNA encoding the stalled peptide can be isolated from the stalled ribosome, which is the basis of ribosome display for peptide screening [7]. The absence of a stop codon or defective mRNA also causes ribosomes to stall [10]. In addition, structured RNA such as hairpins and pseudoknots can also stall the ribosome and induce ribosomal frame-shifting [25]. Ribosomes stalled by mRNAs are assumed to have different resumption strategies, even if the exact mechanism is unclear. Once more structures of stalled ribosome are available, the mechanism of ribosome stalling by mRNA can be understood more completely with this method.

## 3 Conclusion

The ribosome is one of the most complex nanomachines in living cells with a molecular weight of 2.5 MDa. The ribosome has three tRNA binding sites, one mRNA channel, one peptide channel, and multiple binding pockets for translation regulating proteins. Ribosome complexes have been extensively studied with biophysics techniques [18,26–28]. The structures of ribosome complexes in the PDB have increased exponentially since 2000. The complex structure and millions of atoms in the ribosome make visualization and comparison of these super-molecular complexes challenging. Calculation of RNA-protein or protein-protein distance in these ribosome complexes is a good way to find local and global structural changes in these complexes. The RNA-protein distances reflect conformational changes, which can determine what type of molecules interact with the ribosome since the conformational changes of ribosomes alter the binding microenvironment to accommodate these binding molecules such as antibiotics.

However, the binding of some molecules attenuates protein translation and stalls the ribosome. Based on the sources of molecules causing ribosomes to stall, it can be generally divided into three classes: antibiotics, nascent peptide, and mRNA. The strength of translation attenuation depends on the pause of ribosome function and the degree of ribosome stall. All the stalling factors induce global conformational changes. The nascent peptide of SecM causes greater conformational change and exposes L23 for trigger factor binding. The conformational change at the local binding pocket propagates through the rearrangement of rRNA and transfers across the interface of two subunits through tRNA and other inter-subunit contacts. The simultaneous conformational changes correlate the action of multiple functional sites on the ribosome.

Our study provides some insights for understanding the mechanism of translational attenuation and also provides guidance for future biophysical studies. For example, single molecule FRET measures the distance change in the range of 2–8 nm [5]. Thus, the changes of RNA-protein distance in different complexes will provide the guidance for what changes can be studied and where the fluorescent dyes should be labeled to observe a detectable FRET change.

## Figures and Tables

**Table 1:** Four groups of collected PDB entries for *T. thermophilus* and *E. coli* ribosomes

	E. coli	T. thermophilus
30S	3R8N, 3R8O, 3FIH, 2WWL, 2GYB	2HGI, 3D5A, 3FIC, 3OI2, 3OHY, 3OHC, 3OGE
50S	3OAS, 1PNY, 2QBE, 3R8T, 2GYC, 3FIK, 2WWQ	2HGJ, 3D5B, 3FIN, 3OI3, 3OHZ, 3OHJ, 3OH5

**Table 2:** Average sequence identity between *T. thermophilus* and *E. coli* ribosomesa)

S3	S4	S5	S6	S7	S9	S11	S12
52%	50%	49%	31%	53%	54%	55%	74%
S13	S17	S18	L2	L4	L5	L9	L11
58%	49%	52%	59%	37%	57%	40%	59%
L14	L15	L16	L22	L23	L24	L27	L36
69%	40%	58%	56%	44%	47%	51%	63%

a) Identity, number of common nucleotides/subunit size.
